# Synthesising a Fixed-Length Equispaced Linear Array to Produce Dolph–Chebyshev Patterns with Deep Nulls, a Desired Side Lobe Level and Different Beamwidths

**DOI:** 10.3390/s25061685

**Published:** 2025-03-08

**Authors:** Ibai Otero-Gómez, María Elena López-Martín, Juan Antonio Rodríguez-González, Francisco José Ares-Pena

**Affiliations:** 1 Department of Applied Physics, University of Santiago de Compostela, 15782 Santiago de Compostela, Spain; ibai.otero@rai.usc.es (I.O.-G.); ja.rodriguez@usc.es (J.A.R.-G.); 2 Department of Morphological Sciences, University of Santiago de Compostela, 15782 Santiago de Compostela, Spain; melena.lopez.martin@usc.es

**Keywords:** antenna, arrays, pattern synthesis, Dolph–Chebyshev, beamwidth

## Abstract

A method for the synthesis of equally spaced antenna arrays based on the extension of the Orchard–Elliott–Stern technique to radiation patterns with three roots on the negative real axis of the Shelkunoff unit circle is presented. One of these roots is placed on the unit circle and the other two are off the unit circle with coordinates *r* and 1/r. For a desired side lobe level (SLL), the synthesis of patterns with these roots allows for a multiplicity of solutions with different amplitude ratios, obtained by varying the value of r, each of which presents radiation patterns with different beamwidths and directivity, but with two fewer side lobes than the patterns obtained without these restrictions in the roots. The technique has been thoroughly applied to Dolph–Chebyshev patterns of 10, 18 and 40 elements, with a λ/2 spacing and an SLL that guarantees maximum directivity in both cases. This approach ensures the study of examples of all sizes, from small to large. The findings derived from this technique would be applicable in the domain of wireless communications, where the necessity arises for radiation patterns that exhibit low SLL and adaptive beamwidth.

## 1. Introduction

In many applications, antennas constitute a multitude of analogous radiating components. An antenna composed of identical and equally oriented radiating elements is referred to as an array [1]. The use of array antennas presents a number of advantages that are not available with single elements. Firstly, due to the fact that arrays can be made to be of a considerable size, highly directive radiation patterns can be obtained. Secondly, the excitations of the elements can be individually controlled, thereby allowing the desired side lobe topography to be achieved [2].

In the absence of mutual coupling between radiating elements, the total field of the array can be considered to be the sum of the fields radiated by the individual elements. In an array, the total field can be expressed as the product of two factors: the element factor and the array factor. The element factor is equal to the field of a single element, which, due to its small size, presents a wide radiation pattern. The array factor, which is not vector-like, is independent of the characteristics of the radiating element employed and is the sum of directionally weighted phasors. The array factor is determined by the geometric configuration of the radiating elements and their relative excitations. This factor exerts the most influence on the fine structure of the antenna array radiation pattern [3].

Equispaced linear arrays represent the simplest form of arrays, in which the radiating elements are located along a straight line with a constant inter-element spacing between them. It is well known that for these arrays, the relative excitations for a sum pattern with an arbitrary side lobe topography can be uniquely determined. This is achieved through the utilisation of the Schelkunoff unit circle as a design tool [4].

The Dolph–Chebyshev procedure is a well-known approach for synthesising sum radiation patterns using equally spaced arrays, which will constitute our case of study. In addition, a significant amount of research has been conducted on unequally spaced arrays and circular arrays. For instance, as stated in [5], an approach to synthesising unequally spaced arrays is outlined, with the utilisation of an inversion algorithm. In [6], non-uniformly spaced arrays are employed to ensure optimal beam focusing, while ref. [7] details a technique for synthesising unequally spaced arrays with reduced side lobe level peaks. With regard to uniform circular arrays, ref. [8] presents a computationally efficient method for synthesising array patterns with guaranteed maximum side lobe levels. In [9], uniform circular arrays are also synthesised with the purpose of altering the beamwidth. Finally, ref. [10] uses simulated annealing to minimize a cost function.

However, there are some limitations to this method, even in the simplest case of equally spaced arrays. The Dolph–Chebyshev method [2] is unable to independently control the beamwidth and side lobe level for a fixed number of elements and inter-element spacing, due to the fact that the root distribution obtained by this method is unique within the unit circle, as has been previously commented. This limitation is present in the majority of available methods for synthesising equally spaced linear arrays, including the Orchard–Elliott–Stern method [11], which permits the generation of arbitrary sum or difference patterns with user-defined side lobe topography [12]. In [9], a methodology is proposed for the synthesis of Dolph–Chebyshev beam patterns with variable beamwidths. However, in this paper, the topography of side lobes is modified, resulting in a *quasi-Chebyshev* beam pattern. Consequently, the side lobes are not strictly aligned at the same level, and it is not possible to guarantee that a pure Chebyshev pattern will be obtained. The Orchard–Elliott–Stern method [1] is capable of accommodating arbitrary side lobe topography, and a variation in the beamwidth can be obtained using this method as well if one dispenses with the fixed topography hypothesis.

In 1990, Y.U. Kim showed that in planar arrays with a rectangular lattice and circular boundary, the collapsed distribution (i.e., the equivalent linear equispaced array for a constant φ-cut, as described in [1]) at the cut φ=0 degrees is highly tapered and has two roots off the unit circle on the negative real axis with coordinates *r* and 1/r. The same happens with a cut at φ=90 degrees [13]. It is known that, in a polynomial, the product of the roots is the independent term, as will be seen in the Section 2. This relation ensures that the roots do not contribute to this term.

This paper presents an extension of the Orchard–Elliott–Stern method [11] with two roots displaced outside the unit circle on the negative real axis, as previously mentioned. Orchard et al. developed a shaped beam synthesis method that iterates over the roots of the Schelkunoff polynomial [4] by applying a gradient-based technique. This approach perturbs the roots from suitable initial values to minimize ripple and deviation from the desired side lobe levels [2,11]. In most cases, this process guarantees the synthesis of an arbitrary side lobe topography. Since the proposal of the original algorithm, a significant amount of additional work and modifications of the method have been provided [14,15].

The synthesis of patterns with these two roots displaced outside the unit circle and a desired SLL allow for the generation of a multiplicity of solutions, achieved by varying the value of *r*. Each solution presents radiation patterns with different beamwidths and directivity. This allows for a synthesis of patterns with arbitrary beamwidth and other parameters, inside the provided range, achieving a compromise among them. The technique has been applied in detail to Dolph–Chebyshev patterns with an SLL that guarantees maximum directivity for an array comprising a small, a moderate and a medium–large number of elements to ensure the thoroughness of the analysis.

## 2. Materials and Methods

In the case of a general array comprising N+1 elements, the expression for the array factor F(θ,φ) is given by [1]

(1) $$F\left(\theta ,\varphi \right)=\sum_{n=0}^{N}I_{n}e^{jk(x_{n}\sin\theta \cos\varphi +y_{n}\sin\theta \sin\varphi +z_{n}\cos\theta )}$$

where *k* is the wave number, $\left(x_{n},y_{n},z_{n}\right)$ are the coordinates of the n-th array element; $I_{n}$ represents each relative complex excitation; θ and φ are the polar and azimuthal angles, respectively; and, finally, j is the imaginary unit.

A simple yet fundamental example is that of linear arrays [3], wherein equally spaced radiators are arranged in a straight line. If the array were to be positioned along the z-axis with an inter-element spacing of *d*, then Equation (1) would reduce to

(2) $$F\left(\theta \right)=\sum_{n=0}^{N}I_{n}e^{jknd\cos\theta }$$

**Figure 1:**
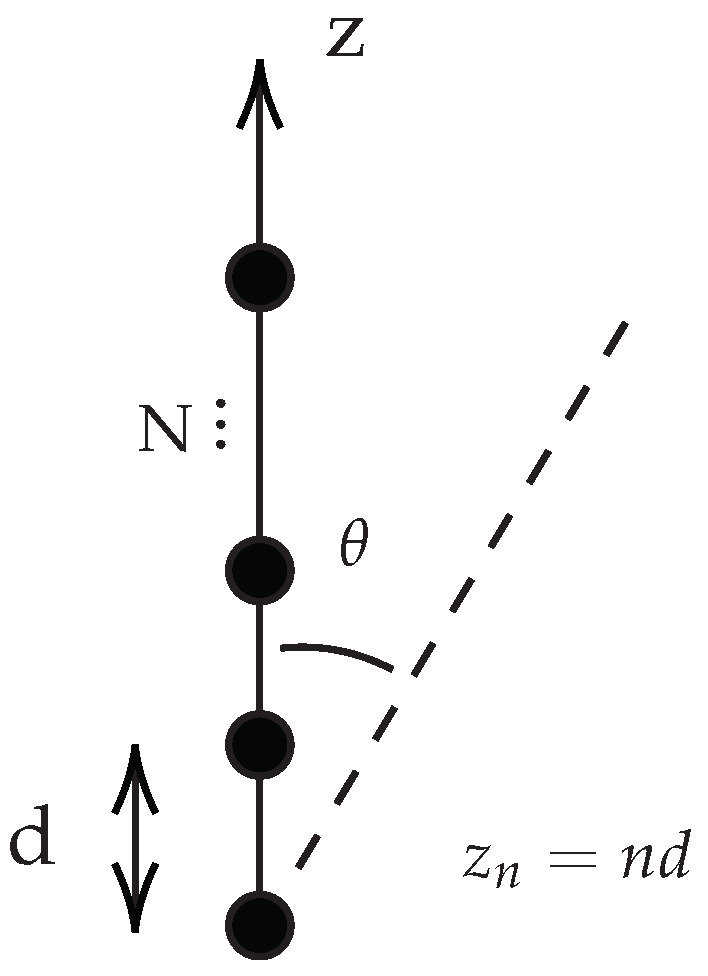
Equispaced linear array placed along the z axis with an inter-element spacing of *d* between elements.

A schematic example is illustrated in Figure 1.

Schelkunoff [4] further simplified expression (2) by means of the following substitution:

$$\omega =e^{j\psi },$$

where ψ=kdcosθ.

In this framework, the array factor is treated mathematically as a polynomial defined in the complex plane. The new variable designated as ω takes values on the unit circle. Since we are dealing with polynomials, it is helpful to write it in terms of its root factorization. Let the roots of the polynomial be $\omega_{n}$, for n=1,⋯,N. The resulting expression for the normalized array factor is thus [12]

(3) $$f\left(\omega \right)=\sum_{n=0}^{N}\frac{I_{n}}{I_{N}}\omega^{n}=\prod_{n=1}^{N}\left(\omega -\omega_{n}\right)$$

By computing the product $\prod_{n=1}^{N}\left(\omega -\omega_{n}\right)$ and comparing both sides of Equation (3), we arrive at the so-called Vieta’s formulas, which relate the excitations $I_{i}$ and the roots $\omega_{n}$:

(4) $$\left\{\begin{array}{cc} \frac{I_{N-1}}{I_{N}} & ={\left(-1\right)}^{1}\left(\omega_{1}+\omega_{2}+\omega_{3}+...+\omega_{N}\right)\\ \frac{I_{N-2}}{I_{N}} & ={\left(-1\right)}^{2}\left(\omega_{1}\cdot \omega_{2}+\omega_{1}\cdot \omega_{3}+...+\omega_{N-1}\cdot \omega_{N}\right)\\ \; & \vdots \\ \frac{I_{j}}{I_{N}} & ={\left(-1\right)}^{N-j}\left(\omega_{1}\cdot \omega_{2}\cdot \omega_{3}\cdots \omega_{j}+...+\omega_{N-j+1}\cdot \omega_{N-j+2}\cdots \omega_{N}\right)\\ \; & \vdots \\ \frac{I_{1}}{I_{N}} & ={\left(-1\right)}^{N-1}\left(\omega_{1}\cdot \omega_{2}\cdot \omega_{3}\cdots \omega_{N-1}+...+\omega_{2}\cdot \omega_{3}\cdot ...\omega_{N}\right)\\ \frac{I_{0}}{I_{N}} & ={\left(-1\right)}^{N}\left(\omega_{1}\cdot \omega_{2}\cdot \omega_{3}\cdots \omega_{N}\right) \end{array}\right.$$

These equations have previously been employed in the context of array pattern synthesis. See, for instance, [16].

The issue of array synthesis is now related to the adjustment of roots in the complex plane until the side lobe levels and other desired parameter values are attained. In order to synthesise a desired pattern for an antenna, it is common practice to employ the Orchard–Elliott–Stern method [11]. This algorithm iterates over the roots of Schelkunoff’s unit circle in order to obtain the appropriate excitations for the pattern. We will now present an extension of the Orchard–Elliott–Stern method in which some roots have been fixed beforehand in specific spots and discuss its advantages and disadvantages. The following lines describe the setup in detail.

**Figure 2:**
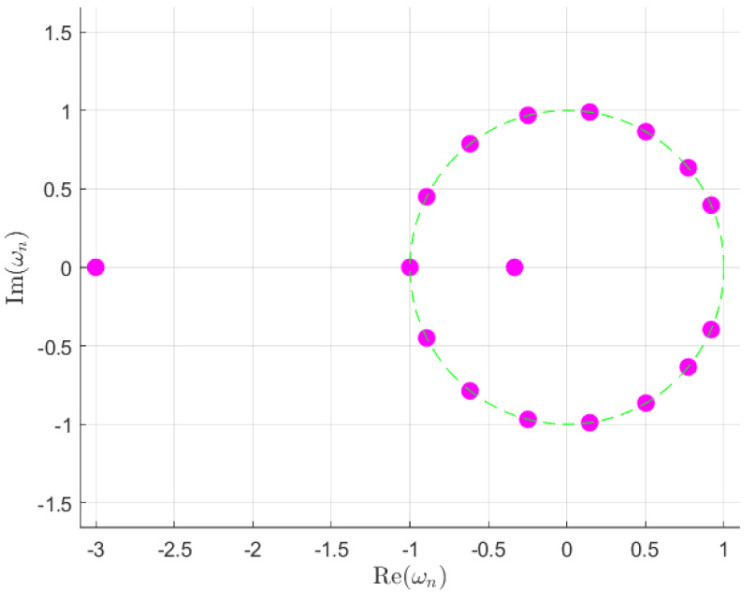
Root position example for r=3 and N=18 elements and the outlined unitary circumference. The SLL for the Dolph–Chebyshev distribution is set to −19 dB.

The initial step is to fix one root at ψ=π in the complex plane. In our model, two roots will be situated on the negative real axis while maintaining a reciprocal relation of *r* and 1/r between their modules. Specifically, one root will be placed within the unit circle while the other will be placed outside. This relation is designed to guarantee that the roots cancel each other out in the expressions displayed in (4). The remaining roots will be distributed around the circumference—thus, $\omega_{n}=e^{jb_{n}}$—ensuring that the pattern is as desired. Additionally, each pair of roots will be mirror images with respect to the *x*-axis, facilitating the formation of symmetric patterns. Consequently, the resulting pattern will be symmetric with respect to the angle θ=π/2. It should be noted that the extension is only applicable to an even number of elements, as this is necessary to place a root in ψ=π. An example of the root positions can be seen in Figure 2.

Our extension of the Orchard–Elliott–Stern method then fixes an *r* value and the corresponding roots, and proceeds to iterate over the remaining roots in the unit circle, ensuring that the desired pattern is achieved. As will be demonstrated, the removal of two roots from the unit circle results in the loss of lobes in the diagram. Therefore, the extension is equipped with the precise number of constraints necessary for the existence and convergence of a solution. In order to plot the figures of the *r* dependence, the algorithm performed 800 syntheses for different *r* values and constructed graphs through polynomial regression of the resulting data points. The range from which *r* is chosen is explained in each subsection. The patterns were computed with 30,000 points. Finally, it was found that this configuration always yields real and symmetric excitations, provided that the pattern is also symmetric.

If our linear array is constituted by 2N equispaced elements, taking the previous considerations into account, we obtain the following:

(5) $$\begin{array}{c} \begin{array}{cc} F(\omega ) & =I_{2N}\left(\omega +1\right)\left(\omega +r\right)\left(\omega +\frac{1}{r}\right)\prod_{n=1}^{2N-4}\left(\omega -\omega_{n}\right)=I_{2N}\left(\omega +1\right)\left(\omega^{2}+\left(r+\frac{1}{r}\right)\omega +1\right)\prod_{n=1}^{N-2}\left(\omega -e^{jb_{n}}\right)\left(\omega -e^{-jb_{n}}\right)\\ \; & =I_{2N}\left(\omega +1\right)\left(\omega^{2}+\left(r+\frac{1}{r}\right)\omega +1\right)\prod_{n=1}^{N-2}\left(\omega^{2}-\left(2\cos b_{n}\right)\omega +1\right) \end{array} \end{array}$$

where we have assumed that the roots are paired symmetrically, i.e., $b_{n}^{\prime }=-b_{n}$. This is imposed to ensure that we have a real and symmetric pattern. The power pattern, expressed in decibels, and as a function of ψ, can be described as follows:

(6) $$\begin{array}{c} \begin{array}{cc} G & \left(\psi \right)=C_{1}+10\log\left(r^{2}+2r\cos\psi +1\right)+10\log\left(\frac{1}{r^{2}}+\frac{2}{r}\cos\psi +1\right)+10\log\left(2\cos\psi +2\right)+10\sum_{n=1}^{N-2}\log\left(2-2\cos\left(\psi -b_{n}\right)\right)\\ & +10\sum_{n=1}^{N-2}\log\left(2-2\cos\left(\psi +b_{n}\right)\right)=C_{1}+20\log\left(2\cos\psi +r+\frac{1}{r}\right)+10\log\left(2\cos\psi +2\right)+20\sum_{n=1}^{N-2}\log\left(2\cos\psi -2\cos b_{n}\right) \end{array} \end{array}$$

where $C_{1}$ accounts for the normalization constant. It can be observed that a pattern of this nature will yield three distinct solutions. The placement of one of the roots under consideration over another, without alteration to the remaining ones, will result in the same power pattern, albeit with differing excitation distributions.

The usual Orchard–Elliott–Stern method [11] employs an iteration process over the function G in order to obtain a desired pattern *S*. The objective is to minimise the difference between G and S in order to achieve a pattern that will ensure the desired level of side lobes is attained. The process is initiated with the following function, where the variable ψ is held constant:

$$G\left(\psi ,b_{1},...b_{N-2}\right)-S\left(\psi \right)$$

The objective is to optimise the function by employing the gradient.

(7) $$d\left(G-S\right)=\sum_{n=1}^{N-2}\frac{\partial G}{\partial b_{n}}\delta b_{n}$$

It is understood that the variable $\delta b_{n}$ denotes the change that this component of the roots will undergo at each stage of the iterative process. Notice that the roots over the negative real axis will remain fixed. This optimization process will usually lead to the desired pattern after only a few iterations.

Lastly, we shall calculate certain parameters of the array, including the directivity, which is computed as follows [1]:

(8) $$D\left(\theta_{0}\right)=\frac{2F\left(\theta_{0}\right)F^{*}\left(\theta_{0}\right)}{\int_{0}^{\pi }F\left(\theta \right)F^{*}\left(\theta \right)\sin\theta \;d\theta }$$

where $F^{*}$ denotes the complex conjugate of *F*.

It should be noted that this methodology is easily generalized in several ways. Firstly, one can place an arbitrary number of roots outside the unit circle, which increases the number of parameters. In addition, different directions for the roots can be studied.

## 3. Results

In this section, we demonstrate the application of the previously introduced methodology to Dolph–Chebyshev antenna arrays as an illustrative example. This kind of antenna distribution is always designed to provide the highest directivity and the narrowest beamwidth for a given number of elements [2], making it a particularly relevant example for consideration.

For the purposes of this study, we will consider the case d=λ/2, where λ represents the wavelength. The directivity in this case greatly simplifies from (8) to [1]:

(9) $$D=\frac{{\left|\sum_{n=0}^{N}I_{n}\right|}^{2}}{\sum_{n=0}^{N}{\left|I_{n}\right|}^{2}}$$

**Figure 3:**
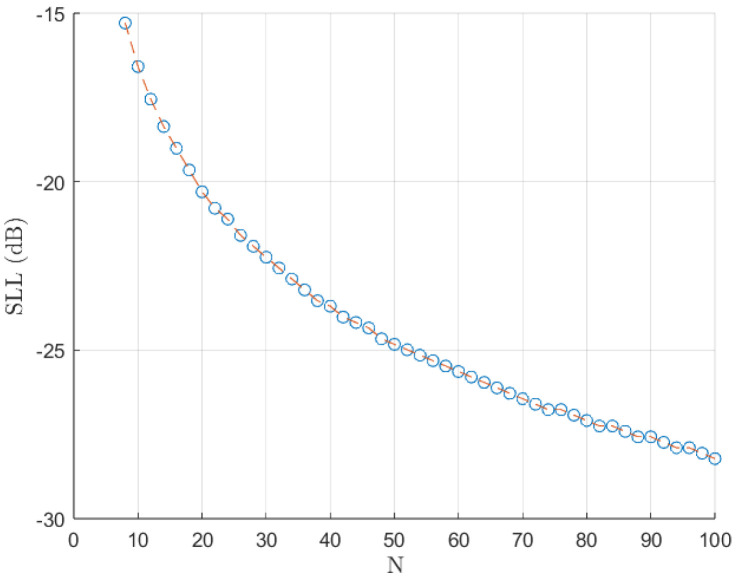
SLL that maximises directivity against the number of elements *N* of the distribution.

Initially, we computed the rounded optimal SLL that maximises the directivity in this kind of pattern (Figure 3). It is beneficial to implement a shuffle of the roots during the synthesis phase to enhance the precision of the solutions, particularly when dealing with extensive arrays in the Orchard–Elliott–Stern method [11]. Henceforth, our discussion will be confined to these specified side lobe levels in the case of Dolph–Chebyshev distributions. Nevertheless, it is not certain whether our method will present optimal directivity for these same SLL values. Therefore, we also computed the optimal SLL values for our extension and for each *r* value. Our findings indicate that the optimal SLL of the modified distribution (the one computed with our extension) appears to consistently fall between the values observed in the N−2 and *N* element distributions for the usual *N* element Orchard–Elliott–Stern synthesis. These values are the ones we will use for the modified distributions.

### 3.1. An 18-Element Array

**Figure 4:**
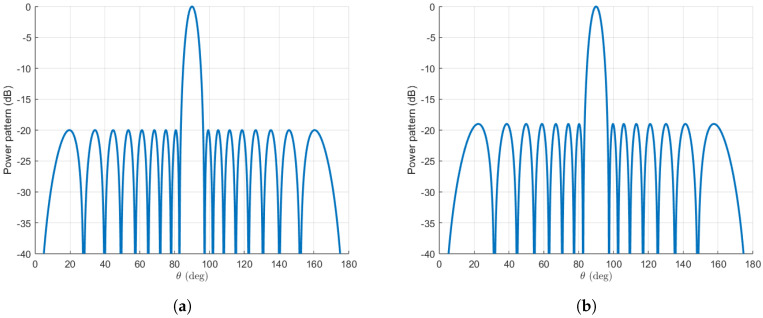
(**a**) Dolph–Chebyshev distribution for 18 elements created with Orchard–Elliott–Stern method. (**b**) Modified Dolph–Chebyshev distribution for 18 elements and r=3.

Our first step is to obtain the pattern for a Dolph–Chebyshev distribution consisting of 18 elements. This is achieved using the Orchard–Elliott–Stern method [11]. The initial pattern is illustrated in Figure 4. The directivity for this pattern amounts to 17.22, 12.36 dBi, considering the SLL that maximizes this value, namely −20 dB. Furthermore, patterns will be synthesised using our extension of the algorithm. For this, an SLL of −19 dB will be used, since it has been found to be an optimal value for directivity. This value does not change substantially with *r* in the range of our study, which is specified below. An illustrative example can be observed in Figure 4 for r=3, which we will soon study in detail.

It can be seen that, as a result of the removal of two roots from the unit circle, the resulting pattern exhibits a reduction in the number of lobes (it has two fewer lobes) compared to the original. This is generally the case when we remove roots from the unit circle [1,13]. Additionally, the diagram exhibits a resemblance to the one synthesised with two fewer elements (the usual Dolph–Chebyshev distribution for N=16 elements). We will see that the reduction in the number of lobes results in an increase in beamwidth, which can be controlled through the parameter *r*, without a significant reduction in directivity.

Our objective now is to examine how the distribution parameters change with respect to *r*. It is important to note at the outset that, by construction, the excitations will always be real and symmetric. As *r* is increased, the dynamic range (amplitude ratios) may be adversely affected, with the minimum occurring at r=1, which corresponds to a triple root located at ψ=π. Nevertheless, a minor peak is observed at the outset of the graph, which should be taken into account when selecting *r*. This is usually the reason for our choice of *r* throughout the article.

**Figure 5:**
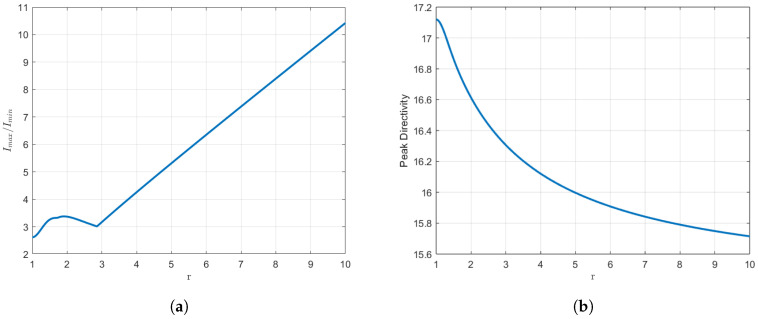
(**a**) Variation in the dynamic range of the distribution with respect to *r*. (**b**) Variation in the directivity of the distribution with respect to *r*.

The values of the *r* parameter are constrained to a range from 1 to 10 because beyond this point, the dynamic range is significantly compromised, with a scaling factor of up to 4 at the upper end of the interval. With regard to directivity, it reaches its maximum at the triple root and subsequently decreases as *r* increases. These phenomena can be observed in Figure 5. In order to undertake a quantitative study of these variations, we will consider a concrete example. For r=3, the dynamic range is observed to increase by 55%, from 2.05 to 3.17, with respect to the usual Orchard–Elliott–Stern synthesis. The directivity is seen to undergo a relatively minor reduction, from 17.22 (12.36 dBi) to 16.31 (12.12 dBi). This implies a decrease of just 5.6%, or 0.24 dBi, from the original configuration. In our range, directivity only declines by 8% in the most unfavourable scenario. A more detailed examination of the impact of the number of elements in these percentages will be undertaken at a later stage.

As commented in the Section 2, a setup like this will yield three distinct solutions by placing one of the roots in consideration on top of another. A similar result to that previously seen is observed when these alternative solutions are subjected to analysis. However, in these cases, there is a greater degree of impact on the dynamic range. Thus, we will refrain from providing extensive commentary on this aspect.

**Figure 6:**
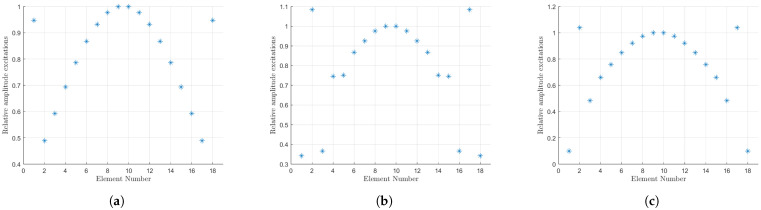
Aperture distribution for the original Orchard–Elliott–Stern synthesis (**a**) and our extension for r=3 (**b**) and r=10 (**c**).

A comparison of these results with the standard Dolph–Chebyshev distribution with 16 elements reveals that our case occupies a position between that of the N=16 and N=18 cases in terms of directivity and excitations. The directivity is constrained to a range between the aforementioned cases. Furthermore, the optimal SLL is shifted closer to that of the N=16 distribution as the value of *r* increases. Finally, if we consider the limit as *r* approaches infinity, we find that the extreme excitations tend to zero and we recover the array for the N=16 case. This is illustrated in Figure 6. The amplitude of the currents has been plotted. These excitations have been normalised, and we have plotted the resulting data for the original Orchard–Elliott–Stern synthesis and for our extension for some *r* values. It can be seen that we achieve a taper in the last term, although the second one is risen.

**Figure 7:**
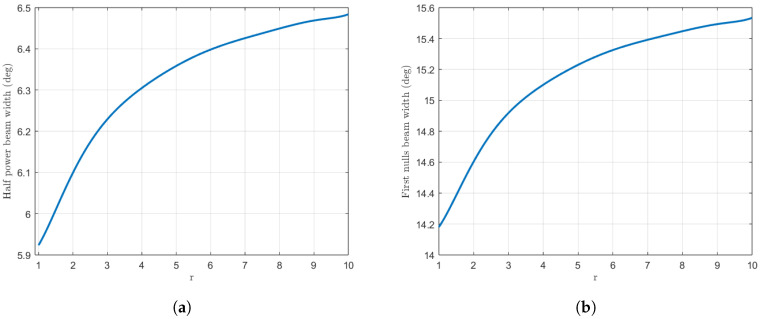
Variation in the beamwidths of the pattern with respect to *r*. In (**a**), the half-power beamwidth is plotted while (**b**) shows the first-null beamwidth.

This approach then offers an intermediary case that presents a number of alternative advantages. Some parameters that can be adjusted by this configuration are the beamwidths of the pattern. Variation in the *r* parameter enables control of the width of the pattern while conserving the side lobe topography. Figure 7 illustrates how the half-power beamwidth (HPBW) and the first-null beamwidth (FNBW) change with respect to the *r* parameter. For an initial synthesis with the Orchard–Elliott–Stern method [11], we obtain a value of 5.98 degrees for the HPBW and 14.47 degrees for the FNBW. For the case r=3, we obtain an HPBW of 6.23 degrees and an FNBW of 14.92 degrees, which represents an increase of 4.2% and 3.1%, respectively. A higher *r* value will imply a more significant rise in these aforementioned values, albeit at the cost of the dynamic range.

In this range, one can achieve from 5.92 up to 6.48 degrees of HPBW and from 14.2 to 15.53 degrees of FNBW.

### 3.2. A 40-Element Array

In order to ensure the thoroughness of our analysis, we will also consider a Dolph–Chebyshev distribution for a larger amount of elements. The methodology will be identical to that previously employed, with the value of *d* kept at λ/2 and the number of elements, *N*, set to 40. In this case, we have found that the optimal SLL for our extension of the method will still be approximated by −24 dB.

**Figure 8:**
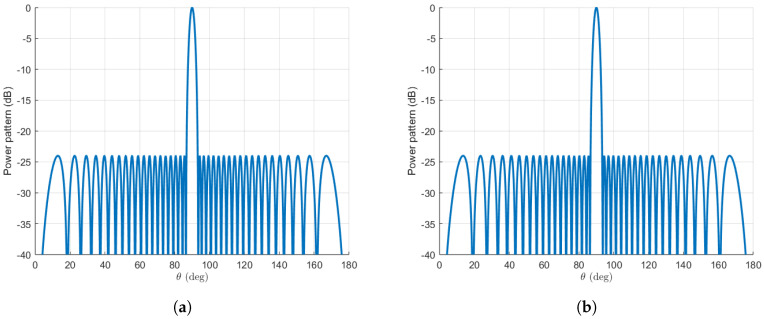
(**a**) Dolph–Chebyshev distribution for 40 elements created with Orchard–Elliott–Stern method. (**b**) Modified Dolph–Chebyshev distribution for 40 elements and r=5. Note that, as in the 18-element example, the last figure has two fewer lobes.

As anticipated, the outcomes pertaining to the configuration of the pattern’s shape remain consistent. Despite the absence of two lobes in our proposed extension, we have succeeded in maintaining the overall shape, which bears resemblance to the 38-element typical Dolph–Chebyshev pattern. As the number of elements increases, the discrepancy becomes increasingly insignificant. The results can be seen in Figure 8.

**Figure 9:**
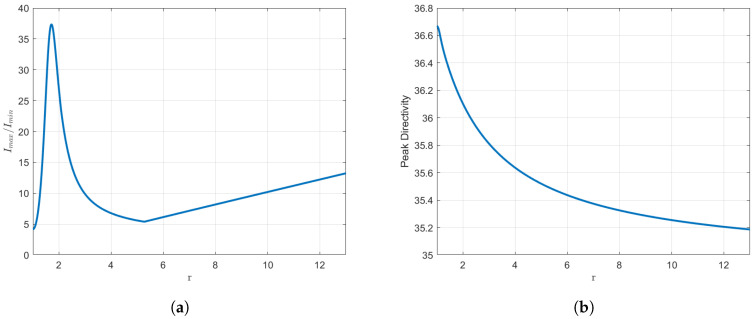

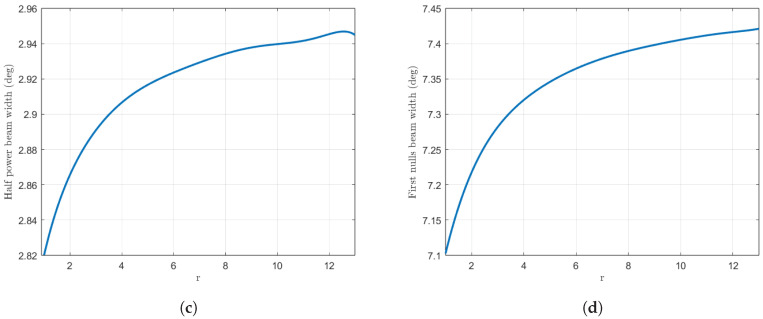
Results for N=40 elements. Different parameters of the distribution are plotted against *r*. (**a**) Variation in the dynamic range. (**b**) Variation in the directivity. (**c**) Variation in the half-power beamwidth. (**d**) Variation in the first-null beamwidth.

We once again examine the parameter dependency with respect to *r* for this case. The *r* parameter may assume values from 1 to 13, as this is approximately the point at which the dynamic range is four times its initial value. The aforementioned data are presented in a graphical format in Figure 9.

As illustrated in Figure 9, the outcomes are comparable to those observed previously. One may highlight that the starting peak in the dynamic range curve is more pronounced. It should also be noted that, from the local minimum in the dynamic range onwards, the rise with *r* remains linear. As observed, the trends remain consistent. Once again, the beamwidths of the pattern can be regulated by modifying the value of the *r* parameter.

In particular, a comparison is made between the r=5 case and the original Orchard–Elliott–Stern synthesis. The increase in dynamic range is now 70%, from 3.28 to 5.60. Moreover, the reduction in directivity amounts only to 3%, or 0.14 dBi, from 36.68 (15.64 dBi) to 35.52 (15.50 dBi). As previously stated, when the highest *r* value is considered, the resulting dynamic range is four times that of the original. In the least favourable scenario, the directivity loss is only 4%.

With regard to the beamwidths of the pattern, in the case of r=5, the HPBW increases from 2.82 degrees to 2.92 degrees, corresponding to a net increase of 3.5%. The first-null beamwidth, on the other hand, rises from a value of 7.09 in the usual synthesis to 7.35, which accounts for an increase of 3.7%. In the most extreme case that we considered, one can achieve a maximum increment of 4.6% and 4.7% respectively. The values of the HPBW range from 2.82 to 2.95 degrees, while the values of the FNBW range from 7.1 to 7.43 degrees, as can be seen in Figure 9.

The results concerning the multiplicity of solutions and the excitations have been found to be exactly analogous to the smaller case. The remaining solutions yield consistent results, albeit with a slight decline in performance. The excitations represent an intermediate case between the 40- and 38-element arrays, with a tendency towards the latter as *r* is increased. Consequently, the excitations provide results that are similar to those observed in Figure 6.

It is clear that the variation in beamwidths is less pronounced in this larger case. This is to be anticipated, given that as the number of elements is increased, the HPBW approaches its minimum value for this kind of distribution. Given that the lobes are all at the same level and that there is a fixed space in which to distribute them, it can be seen that a bound on the HPBW is being approached by increasing *N*. It would therefore be beneficial to examine a case with an even smaller number of elements. In the following lines, we will thus briefly present the results for a distribution comprising only 10 elements.

### 3.3. 10-Element Array

The optimal SLL for the usual Orchard–Elliott–Stern synthesis of the 10-element array has been found to be −17 dB. A good approximation for the optimal SLL of our extension would be −16 dB.

For this Orchard–Elliott–Stern synthesis, we obtained a dynamic range of 1.53. The optimal directivity was found to be 9.81 (9.92 dBi), while the beamwidths were found to be 10.53 degrees and 24.78 degrees for the HPBW and FNBW respectively.

In this case, we let the *r* parameter range from 1 to 5.5, which is again a value of *r* which approximately quadruples the dynamic range compared to the original value. In the edge case, we obtained a maximum value of 12.12 (+15.1%) and 28.38 (+14.5%) degrees for the beamwidths; the loss in directivity was around 13.4%.

As a particular example, if we set r=2.3, the loss in directivity amounts to 7.5% (a value of 9.07, or 9.58 dBi). For the dynamic range, we obtained 2.63. The results for the beamwidths were found to be 11.28 degrees for the HPBW and 26.36 degrees for the FNBW. This implies an increment of 7.1% and 6.4%, respectively.

## 4. Discussion

The application of the technique described makes it possible to obtain numerous solutions by varying the position of the roots outside the unit circle. The synthesis of modified Dolph–Chebyshev distributions with a small and medium number of elements has shown that the variability with the *r* parameter in beamwidth and directivity is greater for the arrays with a small number of elements, but the variation in the amplitude ratios of the excitations is greater for the array with a medium number of elements. In both cases, the solution that gives greater directivity, narrower beamwidths and lower amplitude ratios has the three roots on the unit circle (this corresponds to the case r=1, which is the lower limit in the plots). However, this may not be the case for other distributions or number of elements. This extension has been demonstrated to offer an alternative means of controlling the various parameters of the distribution, without altering the length of the array. We have mainly focused on the variation in the beamwidths. This also allows for distributions with fewer numbers of lobes without drastic alterations of other parameters.

It is apparent that the increments in the beamwidths have been relatively modest. Nevertheless, in the smallest case of 10 elements, a higher increment can be attained. In general, this study indicates that the smaller the array, the higher the observed increments. In the context of Dolph–Chebyshev arrays, it is known that the directivity beamwidth product remains relatively constant for all conditions of side lobe level such that maximum directivity and array length exists [17], which constitutes our case. Consequently, it was anticipated that an increase in beamwidth would be accompanied by a corresponding decrease in directivity. This was found to be the case. For a total of N=10 elements, the loss in directivity was 7.5%, while for N=18 elements, it was 5.6%. For N=40 elements, only 3% was the loss in directivity. These findings were observed for specific values of *r* that were considered within the study, but the conclusion applies in any case.

The proposed method, which relies on precise control of radiation patterns through root placement on the Schelkunoff unit circle, shares conceptual similarities with diffractive neural networks (DNNs). DNNs could potentially be employed to automate or enhance the synthesis process, particularly for complex array configurations or multi-objective optimization scenarios. For instance, DNNs could efficiently explore the solution space and identify optimal root placements and amplitude ratios that satisfy multiple constraints, such as side lobe levels, beamwidth, and directivity. The *r* parameter, along with the various parameters that could be obtained as a result of removing more than a pair of roots from the unit circle, could be the outputs of the aforementioned methods. Recent advances in DNNs [18,19] highlight their potential for wavefront manipulation and diffraction control, which could be directly applicable to the synthesis of antenna arrays.

Our extension can be applied directly to difference (placing the roots in the ψ=0 axis instead) and shaped beam patterns. Similarly, any additional pattern shape could be considered. In addition to the previous examples, we have conducted syntheses with a single root, placed either inside or outside the unit circle, and across a range of ψ-cuts. These outcomes consistently yielded inferior results—sometimes resulting in negative or complex excitations—and thus, they have not been included in this presentation. The issue with a single root is that only one lobe is removed, resulting in a loss of symmetry and a reduction in the method’s effectiveness. Maintaining a fixed root at ψ=π and preserving symmetry would require an odd number of elements, resulting in the loss of control over one lobe. The method subsequently returns complex excitations, with certain parameters being distorted. This can be easily verified using, for example, N=17 elements and only one root inside or outside the unit circle.

If one chooses instead to remove more than a pair of roots from the unit circle, there would be more than one parameter available to control the different pattern variables; even optimization methods could be used to achieve desired arrays, although the beam is broadened as a result of the removal of roots. We have not journeyed very far down this road yet. However, all this suggests that there is considerable scope for further research in this area, extending the method in numerous ways.

## Data Availability

The datasets used and/or analysed during this study are available from the corresponding author upon reasonable request.

## Abbreviations

The following abbreviations are used in this manuscript:

SLL	Side Lobe Level
HPBW	Half-Power Beamwidth
FNBW	First-Null Beamwidth
DNN	Diffractive Neural Network
